# Mortality and Thrombosis in Injured Adults Receiving Tranexamic Acid in the Post-CRASH-2 Era

**DOI:** 10.5811/westjem.2019.4.41698

**Published:** 2019-04-26

**Authors:** Simranjeet Benipal, John-Lloyd Santamarina, Linda Vo, Daniel K. Nishijima

**Affiliations:** Universtiy of California, Davis Medical Center, Department of Emergency Medicine, Sacramento, California

## Abstract

**Introduction:**

The CRASH-2 trial demonstrated that tranexamic acid (TXA) reduced mortality with no increase in adverse events in severely injured adults. TXA has since been widely used in injured adults worldwide. Our objective was to estimate mortality and adverse events in adults with trauma receiving TXA in studies published after the CRASH-2 trial.

**Methods:**

We systematically searched PubMed, Embase, MicroMedex, and ClinicalTrials.gov for studies that included injured adults who received TXA and reported mortality and/or adverse events. Two reviewers independently assessed study eligibility, abstracted data, and assessed the risk of bias. We conducted meta-analyses using random effects models to estimate the incidence of mortality at 28 or 30 days and in-hospital thrombotic events.

**Results:**

We included 19 studies and 13 studies in the systematic review and meta-analyses, respectively. The pooled incidence of mortality at 28 or 30 days (five studies, 1538 patients) was 10.1% (95% confidence interval [CI], 7.8–12.4%) (vs 14.5% [95% CI, 13.9–15.2%] in the CRASH-2 trial), and the pooled incidence of in-hospital thrombotic events (nine studies, 1656 patients) was 5.9% (95% CI, 3.3–8.5%) (vs 2.0% [95% CI, 1.8–2.3%] in the CRASH-2 trial).

**Conclusion:**

Compared to the CRASH-2 trial, adult trauma patients receiving TXA identified in our systematic review had a lower incidence of mortality at 28 or 30 days, but a higher incidence of in-hospital thrombotic events. Our findings neither support nor refute the findings of the CRASH-2 trial but suggest that incidence rates in adults with trauma in settings outside of the CRASH-2 trial may be different than those observed in the CRASH-2 trial.

## INTRODUCTION

Hemorrhage is the primary cause of death in the first 24 hours after trauma and is responsible for 40% of all trauma-related deaths.^1^,^2^ Tranexamic acid (TXA), an antifibrinolytic drug that blocks plasmin-mediated fibrin clot breakdown, attenuates excessive bleeding. In patients undergoing surgery, TXA has been shown to decrease blood product transfusion requirements.^3^,^4^ The success of TXA in the surgical setting led to the CRASH-2 trial, an international, randomized controlled trial of the early administration of TXA to bleeding adult trauma patients.^5^ Compared to placebo, TXA given within three hours of injury, reduced the risk of hemorrhagic death by approximately one-third with no increase in adverse events.^6^

Administering TXA is now considered standard treatment in adults with traumatic bleeding and its use has been implemented worldwide.^7^ The use of TXA for injured adults has been estimated to save 112,000 lives per year worldwide.^8^ Given that the CRASH-2 trial was conducted in primarily developing countries where transfusion practices and identification of adverse events may differ compared to developed countries, we sought to estimate the incidence of mortality and thrombotic events in injured adults in the post-CRASH-2 era.^9^ Our objective was to evaluate the incidence of mortality and adverse events in studies published after the CRASH-2 trial results were published.

## METHODS

### Search Strategy

We searched PubMed, Embase, MicroMedex, and ClinicalTrials.gov for studies that included adult trauma patients who received TXA and reported mortality and/or adverse events (Supplemental File). References of potentially eligible articles identified in the search were further screened for relevant references missed in the database search.

### Inclusion and Exclusion

We included all studies that assessed mortality and/or adverse events in adult trauma patients receiving TXA. We included studies regardless of TXA dosing or clinical setting (e.g., prehospital, military, civilian) and studies that reported only mortality or adverse events. We excluded case reports and review articles, studies that were not trauma-related or that included primarily children, and studies that did not report mortality or adverse events. We also excluded studies that were secondary analyses of the CRASH-2 trial.

### Study Selection

**Figure 1:**
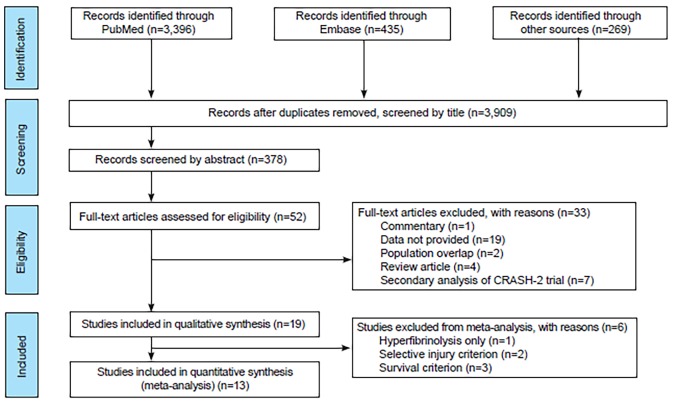
Preferred Reporting Items for Systematic Reviews and Meta-Analyses (PRISMA) diagram depicting selection of studies of articles for review.

We screened studies for inclusion initially by titles, abstracts, and then full texts. Each study title and abstract was reviewed independently by two authors. When consensus could not be reached on screened titles and abstracts, a third reviewer independently adjudicated the discrepancies. Full-text discrepancies were resolved by group consensus during in-person meetings. Prior to independent author screening, we piloted the study selection procedures as a group for several studies to enhance standardization of the selection protocol. Our study selection procedure is reported for in Figure 1 according to the Preferred Reporting Items for Systematic Reviews and Meta-Analyses (PRISMA) (See Figure 1).

### Data Abstraction and Quality Assessment

Two authors independently abstracted data from each of the included studies. We abstracted study characteristics that included the following: year, country, setting (e.g., civilian, military, prehospital), design, inclusion criteria, TXA dosing, and outcomes measured. We abstracted outcome measures including mortality during any time frame, thrombotic events, and other adverse events reported, as well as adverse event definitions provided by the authors of the included study. Extraction was piloted as a group on several studies. We resolved disagreements in extracted data by group discussion and by consensus of all authors.

We assessed the quality of included studies using a quality assessment instrument previously developed by the National Heart, Lung, and Blood Institute.^11^ This instrument included nine points assessing the clarity of the study objective and study population, the sequence of enrollment (consecutive vs. non-consecutive), the comparability of subjects, the clarity of the study intervention and the outcome measures, the adequacy of length of follow-up, the appropriateness of statistical methods, and the clarity of reported results.

### Outcome Measures

Our primary outcomes were mortality at 28 days and in-hospital thrombotic events, as these were outcomes reported in the CRASH-2 trial.^5^ We identified studies that reported mortality at 30 days and thus expanded this outcome to include mortality at both 28 and 30 days. In-hospital thrombotic events for the CRASH-2 trial were defined as any vascular occlusive event including myocardial infarction, stroke, pulmonary embolism (PE) and deep vein thrombosis (DVT). We accepted any definition of an in-hospital thrombotic event as reported by the included studies. Since the majority of the studies reported total thrombotic events rather than the number of patients with thrombosis, we reported on total number of thrombotic events (i.e., if one patient had both a DVT and stroke identified, it would count as two thrombotic events). Secondary outcomes included mortality at 24 hours, in-hospital mortality, and in-hospital PE or DVT.

### Analysis

Prior to pooling the data, we assessed studies for clinical heterogeneity based on study population, setting, design, intervention, and outcome assessment. All authors participated in group discussions to determine which studies should be excluded from the meta-analyses due to significant clinical heterogeneity compared to the other studies. We performed meta-analyses using the random effects model to report incidence with 95% confidence intervals (CI). Statistical heterogeneity was assessed with I^2^ where a value >75% represents considerable heterogeneity.^12^ Forest plots were ordered along the Y-axis by descending sample size.^13^ We did not construct a funnel plot to assess for publication bias as these have been shown to be inaccurate for assessing incidence and may cross the 0 and 100% boundaries.^14^ Statistical analysis was performed using Stata 14.0 (College Station, Texas).

## RESULTS

### Characteristics of Studies

The search strategy yielded a total of 4100 articles. After duplicates were removed and abstracts screened, we assessed 52 full-text articles for eligibility. Of these full-text articles, 33 were excluded. Reasons for exclusion can be found in the PRISMA diagram (Figure 1). Table 1 shows the characteristics of these 19 included studies. We identified 58 studies from ClinicalTrials.gov, 10 of which met our inclusion criteria; however, all were ongoing or have not yet published results.^15^

Six studies were conducted in military hospitals^16^–^21^ and 14 were conducted in civilian hospitals (one study included both military and civilian hospitals).^21^–^34^ One study administered TXA in the prehospital setting.^29^ There were 13 retrospective studies,^16^–^21^,^23^–^25^,^27^,^30^,^31^,^33^ five prospective observational studies,^22^,^26^,^28^,^29^,^32^ and one randomized controlled trial.^34^ Ten studies^22^–^26^,^29^–^32^,^34^ reported administering TXA as given in the CRASH-2 trial (1 gram [g] intravenous [IV] bolus followed by 1 g IV over eight hours); three studies^16^,^20^,^21^ reported giving TXA 1 g bolus (without the maintenance dose), and six studies^17^–^19^,^27^,^28^,^33^ did not report TXA dosing. Three studies^17^,^27^,^34^ administered TXA within eight hours from the time of injury (as done in the CRASH-2 trial), 12 studies^16^,^18^,^21^–^26^,^29^–^32^ administered TXA primarily within three hours from the time of injury, and four studies^19^,^20^,^28^,^33^ did not report the timing of TXA administration.

We did not include six studies in the meta-analyses due to significant clinical heterogeneity compared to the other studies. Reasons for exclusion of these studies were as follows: only included patients with injury by firearm or explosive;^16^ only included patients with hyperfibrinolysis;^24^ only included combat patients who survived 24 hours after injury^27^ or survived to receive treatment at a U.S. military hospital after transport from a combat hospital;^18^,^19^ or only included patients with traumatic brain injury.^34^

### Main Results

We included 13 studies^17^,^20^–^23^,^25^,^26^,^28^–^33^ with 2536 adult trauma patients receiving TXA into the meta-analyses evaluating mortality at 28 or 30 days (five studies);^17^,^25^,^29^,^30^,^33^ in-hospital thrombosis (nine studies);^17^,^21^,^25^,^26^,^29^–^33^ mortality at 24 hours (four studies);^17^,^25^,^29^,^33^ in-hospital mortality (nine studies),^20^–^23^,^26^,^28^,^31^–^33^ and PE and/or DVT (four studies)^17^,^21^,^26^,^29^ (Tables 1 and 2).

**Figure 2:**
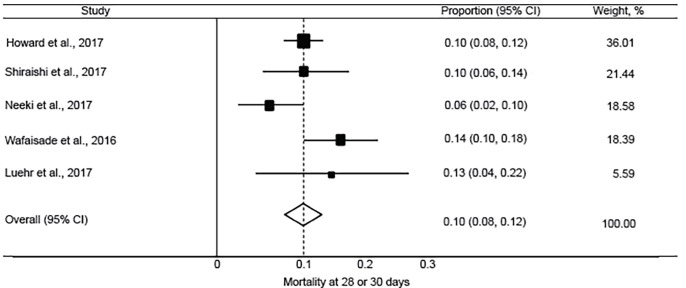
Forest plot of the incidence of mortality at 28 or 30 days after tranexamic acid use in injured adults. *CI*, confidence interval. Chi-square=6.98 p=.137; I^2^=42.7%.

**Figure 3:**
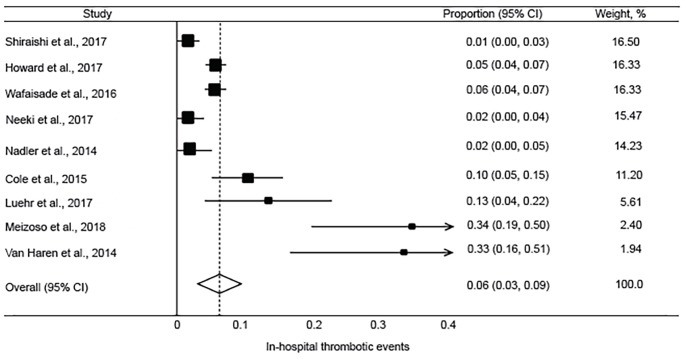
Forest plot of the incidence of in-hospital thrombotic events with tranexamic acid use in injured adults. *CI*, confidence interval. Chi-square=64.74 p<.0001; I^2^=87.6%.

**Figure 4:**
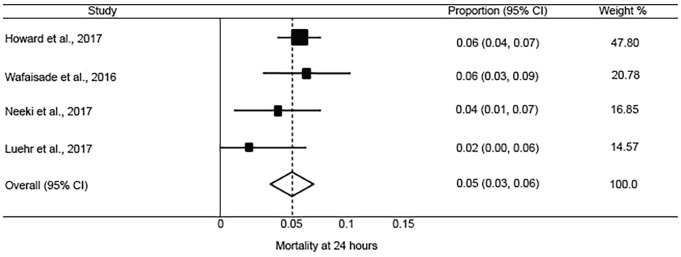
Forest plot of the incidence of mortality at 24 hours after tranexamic acid use in injured adults. *CI*, confidence interval. Chi-square=4.01 p=.260; I^2^=25.2%.

**Figure 5:**
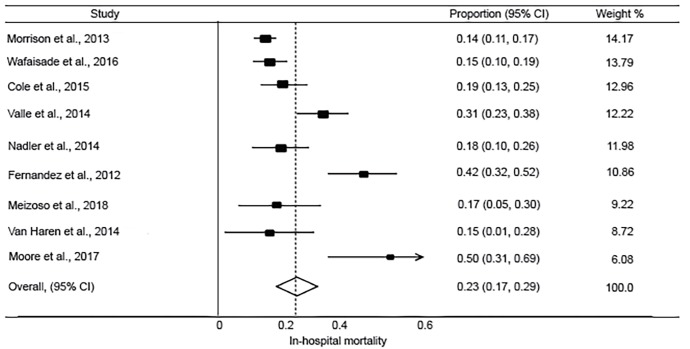
Forest plot of the incidence of in-hospital mortality after tranexamic acid use in injured adults. *CI*, confidence interval. Chi-square=53.35 p<.0001; I^2^=85.0%.

**Figure 6:**
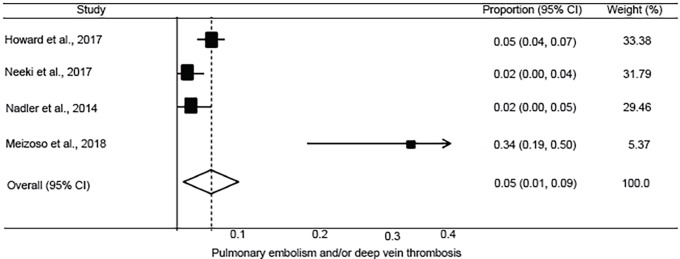
Forest plot of the incidence of pulmonary embolism and/or deep vein thrombosis after tranexamic acid use in injured adults. *CI*, confidence interval. Chi-square=23.19 p<.0001; I*^2^*=87.1%.

The pooled incidence of mortality at 28 or 30 days was 10.1% (95% CI, 7.8 to 12.4%; I^2^ = 42.7%) (Figure 2). This was lower than reported in the CRASH-2 trial, which had an incidence of mortality at 28 days of 14.5% (95% CI, 13.9 to 15.2%) in patients receiving TXA. The pooled incidence of in-hospital thrombotic events was 5.9% (95% CI, 3.3 to 8.5%; I^2^ = 87.6%) (Figure 3). This was higher than reported in the CRASH-2 trial, which had an incidence of in-hospital thrombotic events of 2.0% (95% CI, 1.8 to 2.3%). The pooled incidences of the secondary outcomes of mortality at 24 hours, in-hospital mortality, and PE and/or DVT are reported in Figures 4 to 6.

Our quality assessment suggested concerns regarding non-consecutive patient enrollment (six studies)^18^,^19^,^26^,^27^,^31^,^32^ and an unclear description of the intervention (six studies).^17^–^19^,^27^,^28^,^33^ See Supplemental File, eTable for complete quality assessments of the studies.

## DISCUSSION

Our study demonstrated some interesting findings, particularly in comparison to the CRASH-2 trial. In our study, trauma centers demonstrated a wide variation of TXA administration including dosing (bolus vs bolus + maintenance), total bolus dose (1 g vs 2 g), and timing (within three hours vs eight hours from injury). The CRASH-2 trial administered TXA as a 1 g IV bolus infusion over 10 minutes and a 1 g maintenance infusion over eight hours within eight hours from the time of injury. The varying timing of TXA adminstration noted in our study is likely a result of an exploratory analysis that demonstrated increased benefit in preventing hemorrhagic death with earlier TXA administration given within one hour.^6^ The greatest benefit occurs when TXA is given within <1 hour from injury, diminished benefit if given within one to three hours from injury, and no benefit if given after three hours from injury.^6^ In contrast to the CRASH-2 trial, three studies primarily administered only a bolus dose of TXA as opposed to a bolus dose with a subsequent maintenance dose.^16^,^20^,^21^ This may contribute to different thrombosis and mortality rates. Current clinical trials are evaluating different TXA doses in injured patients.^15^

Compared to the CRASH-2 trial, our pooled results demonstrated a lower incidence of mortality at 28 or 30 days and a higher incidence of in-hospital thrombotic events. We do not conclude from our findings that the effectiveness and harm of TXA is different than what was demonstrated in the CRASH-2 trial, as our study included primarily observational studies. Our results instead suggest that the incidence rates observed in settings outside of the CRASH-2 trial might be different than what was observed in the CRASH-2 trial. This is particularly true for the incidence of thrombotic events, where our pooled results demonstrated a higher incidence of thrombotic events than what was observed in the CRASH-2 trial. The incidence rates reported in our included studies are likely biased towards under-reporting thrombotic events due to the high proportion of retrospective studies (more difficult to identify thrombotic events) and less-comprehensive definition of thrombotic event (often did not include arterial thromboses such as myocardial infarction or stroke) compared to the CRASH-2 trial.

It is unclear why there are differences in thrombotic events seen in the CRASH-2 trial and our study. It is possible that the injury severity of the two study populations is different. We were unable to compare overall patient characteristics of our included studies with those of the CRASH-2 trial. It is also possible that sites included in the CRASH-2 trial screened less for thrombotic events compared to sites included in our study. Other large trauma clinical trials enrolling similarly injured populations have also reported higher thrombotic event rates compared to the CRASH-2 trial.^35^–^37^ Several ongoing trauma clinical trials evaluating TXA should provide additional insight into the incidence of thrombotic events in this population.^15^

Future studies evaluating TXA use in patients with hemorrhagic injuries may consider work to identify patients where the potential efficacy of TXA use is maximized and exposure to harm is minimized. Identification may be based on clinical characteristics, transport time or modality, or laboratory measurements such as thromboelastography.^38^,^39^

## LIMITATIONS

Our results should be interpreted in the context of some limitations. First, the included studies demonstrated clinical heterogeneity, limiting the numbers of studies that could be included in the meta-analyses. Second, studies had varying definitions of in-hospital thrombosis, which may lead to differences in reported incidence rates. Third, the majority of the studies were retrospective, and this may result in less accurate data abstraction compared to prospective studies.^40^ This limitation is more relevant for the thrombosis outcome measure, which may be difficult to ascertain from retrospective chart review, than for the mortality outcome measure, which is easy to ascertain regardless of study design. Fourth, the chart abstractors were not blinded to the study hypotheses. This may have led to biases during study selection and data abstraction. Finally, the incidence of thrombotic events is ideally measured with the number of patients with any thrombotic event as the numerator and the total number of patients as the denominator. However, since included studies primarily reported total number of thrombotic events, we used the total number of thrombotic events as the numerator and the total number of patients as the denominator for calculating incidence.

## CONCLUSION

Compared to the CRASH-2 trial, adult trauma patients receiving TXA identified in our systematic review had a lower incidence of mortality at 28 or 30 days, but a higher incidence of in-hospital thrombotic events. Our findings neither support nor refute the findings of the CRASH-2 trial. They merely suggest that incidence rates observed in settings outside of the CRASH-2 trial may be different than those observed in the CRASH-2 trial.

## Supplementary Information



## Figures and Tables

**Table 1:** Characteristics of included studies.

Study (author, year)	Setting	Design	Patients	TXA dosing	Outcomes measured
CRASH-2 collaborators, 2010^a^	Civilian (Multinational)	RCT, May 2005 –March 2010	10,060 patients, adults with (SBP <90 mmHg or heart rate >110 bpm) or at risk of significant hemorrhage	1 g IV bolus and 1 g IV maintenance within 8 h of injury	Mortality at 28 days, vascular occlusive events, surgical intervention
Aedo-Martin et al., 2016	Military (Afghanistan)	Retrospective, March 2014–May 2014	10 patients, with injury by firearm or explosive	1 g (80%) or 2 g (20%) IV bolus within 3 h of injury	Survival 15 days after discharge, and VTE
Cole et al., 2015^b^	Civilian (UK)	Prospective, October 2010–October 2012	160 patients, >15 y with ISS >15 and admitted to the ICU	1 g IV bolus and 1 g IV maintenance within 3 h of injury	<48 h and >48 h mortality, organ failure, infection, VTE, stroke, myocardial infarction
Fernandez et al., 2012^b^	U.S. Level 1 trauma center	Retrospective, March 2011–July 2012	100 patients, received TXA for trauma	1 g IV bolus and 1 g IV maintenance within 3 h of injury	In-hospital mortality
Harvin et al., 2015	U.S. Level 1 trauma centers	Retrospective, September 2009–September 2013	98 patients, evidence of hyperfibrinolysis (LY30 ≥3%)	1 g IV bolus and 1 g IV maintenance within 3 h of injury	Mortality (in-hospital and 24 h), thrombotic complications
Howard et al., 2017^b^	Military (Afghanistan)	Retrospective, October 2010 –March 2014	849 patients, combat injured, admitted to a medical treatment facility, and received at least one unit of blood	Dose NR, <1 h from time of injury (62.3%), 1–3 h (26.5%), >3 h (10.7%)	24 h, 48 h, 30 days mortality, PE, DVT
Johnston et al., 2018	Military (Afghanistan/Iran)	Retrospective, 2011–2015	146 patients, combat injured and treated at Walter Reed National Military Medical Center	Dose NR, ≤3 h from time of injury (95.9%), >3 h (4.1%)	Mortality, VTE
Lewis et al., 2016	Military (Afghanistan/Iran)	Retrospective, June 2009–December 2013	335 patients, combat injured, treated at military hospital, and received blood products	Dose NR	Infection within 30 days of injury, mortality
Luehr et al., 2017^b^	U.S. Level 1 trauma center	Retrospective, 2013–2016	53 patients, survived >8.5 hours (minimum time required to receive full TXA dose), received at least a single blood product, and heart rate >120 bpm or SBP <90 mmHg	1 g IV bolus and 1 g IV maintenance within 3 h of injury	Mortality
Meizoso et al., 2018^b^	U.S. Level 1 trauma center	Prospective, August 2011–January 2015	35 patients, admitted to the ICU and had TEG completed	1 g IV bolus and 1 g IV maintenance within 3 h of injury	Acute kidney injury, acute lung injury, hyperbilirubinemia, hemodynamic instability requiring vasopressors, VTE, mortality, hospital LOS, ICU free days
Milligan et al., 2016	U.S. Level 2 trauma center	Retrospective, June 2013–June 2016	65 patients, received TXA for trauma and survived >24 h after injury	Dose NR, <3 h from time of injury (49.2%), >3 h (53.8%)	In-hospital mortality
Moore et al., 2017^b^	U.S. Level 1 trauma center	Prospective, 2014–2016	26 patients, >18 years, highest trauma activation, and NISS >15	Dose NR	In-hospital mortality
Morrison et al., 2013^b^	Military (Afghanistan)	Retrospective, March 2006–March 2011	406 patients, combat injured, admitted to medical treatment facility, and received at least one unit of blood	1 g IV bolus, followed by further doses at clinician’s discretion	In-hospital mortality
Nadler et al., 2014^b^	Civilian and Military (Israel)	Retrospective, December 2011–August 2013	94 patients, received TXA for trauma	1 g IV bolus, <1 h (83.0%), ≥1 h (17.0%)	Mortality, thromboembolisms
Neeki et al., 2017^b^	Prehospital (U.S.)	Prospective, June 2014–March 2015	128 patients, ≥18 y with signs and symptoms of hemorrhagic shock	1 g IV bolus (prehospital) and 1 g IV maintenance within 3 h of injury	Mortality, adverse events, total blood product transfused
Shiraishi et al., 2017^b^	Civilian (Japan)	Retrospective, January 2012–December 2012	250 patients, ISS > 15	1 g IV bolus and 1 g IV maintenance within 3 h of injury	28 day mortality, cause specific mortality
Valle et al., 2014^b^	U.S. Level 1 trauma center	Retrospective, August 2009–January 2013	150 patients, underwent emergency operative intervention directly from the resuscitation area	1 g IV bolus and 1 g IV maintenance within 3 h of injury	Mortality, fluid requirements, length of stay, ICU days
Van Haren et al., 2014^b^	U.S. Level 1 trauma center	Prospective, August 2011–March 2013	27 patients, trauma ICU admission, risk assessment profile for VTE ≥10 and an indwelling CVC or arterial catheter	1 g IV bolus and 1 g IV maintenance within 3 h of admission	Mortality, VTE, LOS, ICU days
Wafaisade et al., 2016^b^	Civilian (Germany)	Retrospective, January 2012–Dec 2014	258 patients, potentially life threatening injury and treatment at a trauma center	Dose NR	Mortality, VTE, sepsis, multiorgan failure, death, LOS
Yutthakasemunt et al., 2013	Civilian (Thailand)	RCT, Oct 2008 to Aug 2009	120 patients, moderate to severe TBI	1 g IV bolus and 1 g IV maintenance within 8 h of injury, mean time from injury 6.6 h (SD 1.7 h)	Mortality, stroke, PE, DVT, GI bleed, unfavorable GOS score outcome, progressive intracranial hemorrhage, blood transfusion, neurosurgical intervention

*TXA*, tranexamic acid; *SBP*, systolic blood pressure; *bpm*, beats per minute; *RCT*, randomized controlled trial; *ICU*, intensive care unit; *LY30*, lysis time at 30 minutes (thromboelastography); *ISS*, injury severity score; *NISS*, new injury severity score; *VTE*, venous thromboembolic event; *CVC*, central venous catheter; *TBI*, traumatic brain injury; *NR*, not reported, *IV*, intravenous; *SD*, standard deviation; *PE*, pulmonary embolism; *DVT*, deep vein thrombosis; *LOS*, length of stay; *GI*, gastrointestinal; *GOS*, Glasgow Outcome Scale; ; *mmHg*, millimeters of mercury; *TEG*, thromboelastography; *g*, grams; *RCT*, randomized controlled trial.

a included as a reference;

b included in the meta-analyses

**Table 2:** Reported mortality and thrombotic complications of included studies.

Study (author, year)	Mortality at 24 h, n (%)	Mortality at 28 or 30 d, n (%)	Mortality, in-hospital, n (%)	Crude thrombosis, n (%)	PE or DVT, n (%)
CRASH-2 collaborators, 2010^a^		1,463 (14.5)		204 (2.0)^c^	112 (1.1)
Aedo-Martin et al., 2016	0 (0)		0 (0)	0 (0)	0 (0)
Cole et al., 2015^b^			30 (18.8)	16 (10)^d^	
Fernandez et al., 2012^b^			42 (42)		
Harvin et al., 2015	33 (34)		39 (40)	6 (6.3)	3 (3.3)^j^
Howard et al., 2017^b^	47 (5.5)	82 (9.7)		45 (5.3)^e^	45 (5.3)
Johnston et al., 2018			1 (0.7)	50 (34.2)^f^	
Lewis et al., 2016			10 (3.0)		
Luehr et al., 2017^b^	1 (1.9)	7 (13.5)		7 (13.2)^f^	
Meizoso et al., 2018^b^			6 (17.1)	12 (34.3)^e^	12 (34.3)
Milligan et al., 2016			5 (7.7)		
Moore et al., 2017^b^			13 (50.0)		
Morrison et al., 2013^b^			57 (14.0)		
Nadler et al., 2014^b^			17 (18.1)	2 (2.4)^g^	2 (2.4)^g^
Neeki et al., 2017^b^	5 (3.9)	8 (6.3)		2 (1.6)^h^	2 (1.6)^h^
Shiraishi et al., 2017^b^		25 (10.0)		3 (1.2)^f^	
Valle et al., 2014^b^			46 (30.7)		
Van Haren et al., 2014^b^			4 (14.8)	9 (33.3)^f^	
Wafaisade et al., 2016^b^	15 (5.8)	36 (14.7)	38 (14.7)	4 (5.6)^i^	
Yutthakasemunt et al., 2013			12 (10.0)	0 (0)	0 (0)

*h*, hours; *d*, days; *PE*, pulmonary embolism; *DVT*, deep vein thrombosis; *MI*, myocardial infarction.

a included as a reference;

b included in the meta-analyses;

c PE, DVT, MI, or stroke;

d VTE, MI, or stroke;

e PE or DVT;

f VTE only;

g out of 83 patients;

h DVT only;

i out of 71 patients;

j PE only.
