# Supplementary material for: Mortality and Thrombosis in Injured Adults Receiving Tranexamic Acid in the Post-CRASH-2 Era
Source: West J Emerg Med. 2019 Apr 26;20(3):443–53. doi: 10.5811/westjem.2019.4.41698 (PMC6526890; doi:10.5811/westjem.2019.4.41698)
Supplement: Supplementary file 1 [file wjem-20-443-s001.docx]

**Appendix.** Search Strategy (conducted 01/23/2018)

PubMed

Use the Advanced Search Builder, and search All Fields.

1st field: “Tranexamic acid” [MESH] OR “antifibrinolytic agents” [MESH] OR “tranexamic acid” [text] OR txa [text]

Boolean logic for 2nd field: AND

2nd field: trauma [text] OR “wounds and injuries” [MESH] OR “multiple trauma” [MESH] OR “hemorrhage” [MESH] OR “exsanguination” [MESH] OR “mortality” [MESH] OR “hospital mortality” [MESH] OR “thrombosis” [MESH] OR “venothrombosis” [MESH]

Embase

Apply the following limitations to each search:

Human (Found under Quick limits)

English (Found under Language)

Also, apply following age restrictions found under Age (includes all categories that fit inclusion of adult as 18 or older):

Young adult: 18 to 24 years old

Adult: 18 to 64 years old

Middle aged: 45 to 64 years old

Aged: 65+ years old

Very elderly: 80+ years

After applying these limitations for each search option, search the following:

“tranexamic acid” OR “TXA”

“trauma” OR “injury”

Combine these two search options.

MicroMedex

Search tranexamic acid.

Go to References (use In-Depth Answers).

ClinicalTrials.gov

Search tranexamic acid AND trauma

**eTable.** Quality Assessment of Included Studies.

|  | Quality Assessment ^a^ | | | | | | | | |
| --- | --- | --- | --- | --- | --- | --- | --- | --- | --- |
| Study (author, year) | Was the study question or objective clearly stated? | Was the study population clearly and fully described, including a case definition? | Were the cases consecutive? | Were the subjects comparable? | Was the intervention clearly described? | Were the outcome measures clearly defined, valid, reliable, and implemented consistently across all study participants? | Was the length of follow-up adequate? | Were the statistical methods well-described? | Were the results well-described? |
| Aedo-Martin et al. 2016 | Y | Y | Y | Y | Y | Y | Y | Y | Y |
| Cole et al. 2015 ^b^ | Y | Y | Y | Y | Y | Y | Y | Y | Y |
| Fernandez et al. 2012 ^b^ | Y | Y | Y | CD | Y | Y | CD | CD | Y |
| Harvin et al. 2015 | Y | Y | Y | N | Y | Y | Y | Y | Y |
| Howard et al. 2017 ^b^ | Y | Y | Y | Y | N | Y | Y | Y | Y |
| Johnston et al. 2018 | Y | Y | N | N | N | N | Y | Y | Y |
| Lewis et al. 2016 | Y | Y | N | Y | N | Y | Y | Y | Y |
| Luehr et al. 2017 ^b^ | Y | Y | Y | Y | Y | N | Y | Y | Y |
| Meizoso et al. 2018 ^b^ | Y | Y | N | Y | Y | Y | Y | Y | Y |
| Milligan et al. 2016 | Y | Y | N | Y | N | Y | Y | N | Y |
| Moore et al. 2017 ^b^ | Y | Y | Y | Y | N | Y | Y | Y | Y |
| Morrison et al. 2013 ^b^ | Y | Y | Y | Y | Y | Y | Y | Y | Y |
| Nadler et al. 2014 ^b^ | Y | Y | Y | N | Y | Y | Y | Y | Y |
| Neeki et al. 2017 ^b^ | Y | Y | Y | Y | Y | Y | Y | Y | Y |
| Shiraishi et al. 2017 ^b^ | Y | Y | Y | Y | Y | Y | Y | Y | Y |
| Valle et al. 2014 ^b^ | Y | Y | Y | Y | Y | Y | Y | Y | Y |
| Van Haren et al. 2014 ^b^ | Y | Y | N | Y | Y | Y | Y | Y | Y |
| Wafaisade et al. 2016 ^b^ | Y | Y | N | Y | N | Y | Y | Y | Y |
| Yutthakasemunt et al. 2013 | Y | Y | Y | Y | Y | Y | Y | Y | Y |

^a^ - Y = yes, N = no, CD = cannot determine

^b^ – included in the meta-analyses
